# Prospective Study to Assess Long‐Term Outcomes of Chelator‐Based Treatment With Trientine Dihydrochloride in Patients With Wilson Disease

**DOI:** 10.1002/jgh3.70114

**Published:** 2025-03-17

**Authors:** Isabelle Mohr, Carlot Kruse, Verena Aliane, Karl Heinz Weiss

**Affiliations:** ^1^ Internal Medicine IV, Department of Gastroenterology University Hospital Heidelberg Heidelberg Germany; ^2^ Prescription Medicines Business Unit Univar Solutions BV Rotterdam the Netherlands; ^3^ Department of Internal Medicine Salem Medical Center Heidelberg Germany

**Keywords:** hepatic disease, neurologic disease, trientine dihydrochloride, Unified Wilson Disease Rating Scale, Wilson disease

## Abstract

**Background and Aims:**

Wilson disease is an inherited disorder of copper metabolism affecting mainly the liver and brain. Trientine dihydrochloride (TETA‐2HCl) is approved for the treatment of Wilson disease in patients (≥ 5 years) intolerant to D‐penicillamine therapy. This study assessed the long‐term outcomes of treatment with TETA‐2HCl in Wilson disease patients.

**Methods:**

In this Phase 2, prospective study, patients continued their treatment of TETA‐2HCl 300 mg (200 mg trientine base) for 12 months (July 2015–April 2017) at one center in Germany. Primary outcomes were the safety and efficacy of TETA‐2HCl treatment; Biomarkers of copper metabolism; and the course of hepatic and neurologic disease.

**Results:**

Overall, 51 patients contributed data. Almost all patients (50 [98.0%]) were considered responders (rating of ≤ 4 at the 12‐month visit); Unified Wilson Disease Rating Scale scores improved throughout the study from a mean (standard deviation) of 11.3 (24.31) at Baseline to 8.8 (22.86) at Month 12. Biochemical assessments of liver parameters (transaminases, liver synthesis) as well as markers of copper metabolism (24‐h urinary copper, non‐ceruloplasmin bound copper (NCC)) showed improved or stable disease throughout the study. Treatment‐emergent adverse events were reported in five (9.6%) patients. No patients withdrew from treatment due to adverse events, and no serious adverse events were considered to be treatment‐related.

**Conclusions:**

This study demonstrated that treatment with TETA‐2HCl was effective and well tolerated in hepatic and neurologic disease manifestations. Additionally, improvement in neurological symptoms was reported throughout the trial, suggesting that improvements may be reported for an extended period after initiation of therapy.

**Trial Registration:** NCT02426905

## 1 Introduction

Wilson disease (WD) is a rare, autosomal recessive disorder, affecting approximately 1 in 30 000 people [1]. The disorder results from mutations in the ATP7B gene (a copper‐transporting ATPase) causing toxic accumulations of copper (Cu), particularly in the liver and brain [2, 3]. Patients with WD present with a range of clinical manifestations, including hepatic, neurological, and psychiatric symptoms [2, 4, 5].

Treatment for WD aims to regain normal Cu balance through the removal of accumulated Cu and/or the prevention of additional Cu uptake [4, 6, 7]. Cu chelators such as D‐penicillamine or trientine increase Cu excretion, and trientine also blocks Cu absorption in the GI‐tract [8, 9]. In Europe, two different formulations of trientine (TETA‐4HCl and TETA‐2HCl) are approved for the treatment of WD in patients who are intolerant to D‐penicillamine therapy, aged 5 years or older [4, 10, 11]. A pharmacokinetic study on the safety and tolerability of the two oral formulations of trientine available (trientine tetrahydrochloride [TETA‐4HCl] tablets vs. trientine dihydrochloride [TETA‐2HCl] capsules) reported more rapid absorption of trientine TETA‐4HCl and a greater systemic exposure than TETA‐2HCl in healthy subjects. However, the elimination rate and terminal half‐life were similar in both formulations [12].

Clinical studies in healthy volunteers and patients with WD have demonstrated that trientine is effective and generally well tolerated, and may have fewer adverse effects than D‐penicillamine [4, 6, 13, 14, 15, 16]. A randomized, open‐label, non‐inferiority, Phase 3 trial of trientine in comparison to D‐penicillamine in 53 WD patients reported that both treatments were effective and generally well tolerated; however, three serious adverse events (AEs) were reported in the D‐penicillamine group, while none were reported in the trientine group [17]. Additionally, a retrospective analysis of 380 patients with WD that assessed the safety of both D‐penicillamine and TETA‐2HCl therapy reported hepatic improvements in most patients and neurological improvements in over half the patients, regardless of the treatment used [13]. Importantly, fewer trientine treatments were discontinued, with 7.1% of trientine treatments discontinued due to AEs, compared to 28.8% of D‐penicillamine treatments [13]. A recent publication of a retrospective study in 77 patients confirmed the long‐term efficacy and tolerability of TETA‐2HCl in patients with WD who had withdrawn from therapy with D‐penicillamine [14].

AEs related to trientine therapy, although uncommon, include iron deficiency and sideroblastic anemia, dermatitis, and colitis [4, 6, 10]. Neurologic deterioration has also been reported as a rare symptom, especially following treatment initiation [13].

The aim of this study was to prospectively assess the long‐term safety and efficacy of TETA‐2HCl treatment in WD following withdrawal from D‐penicillamine, including markers of Cu metabolism, the course of hepatic and neurologic disease in an open‐label Phase 2 trial.

## 2 Methods

### 2.1 Study Design

**FIGURE 1:**
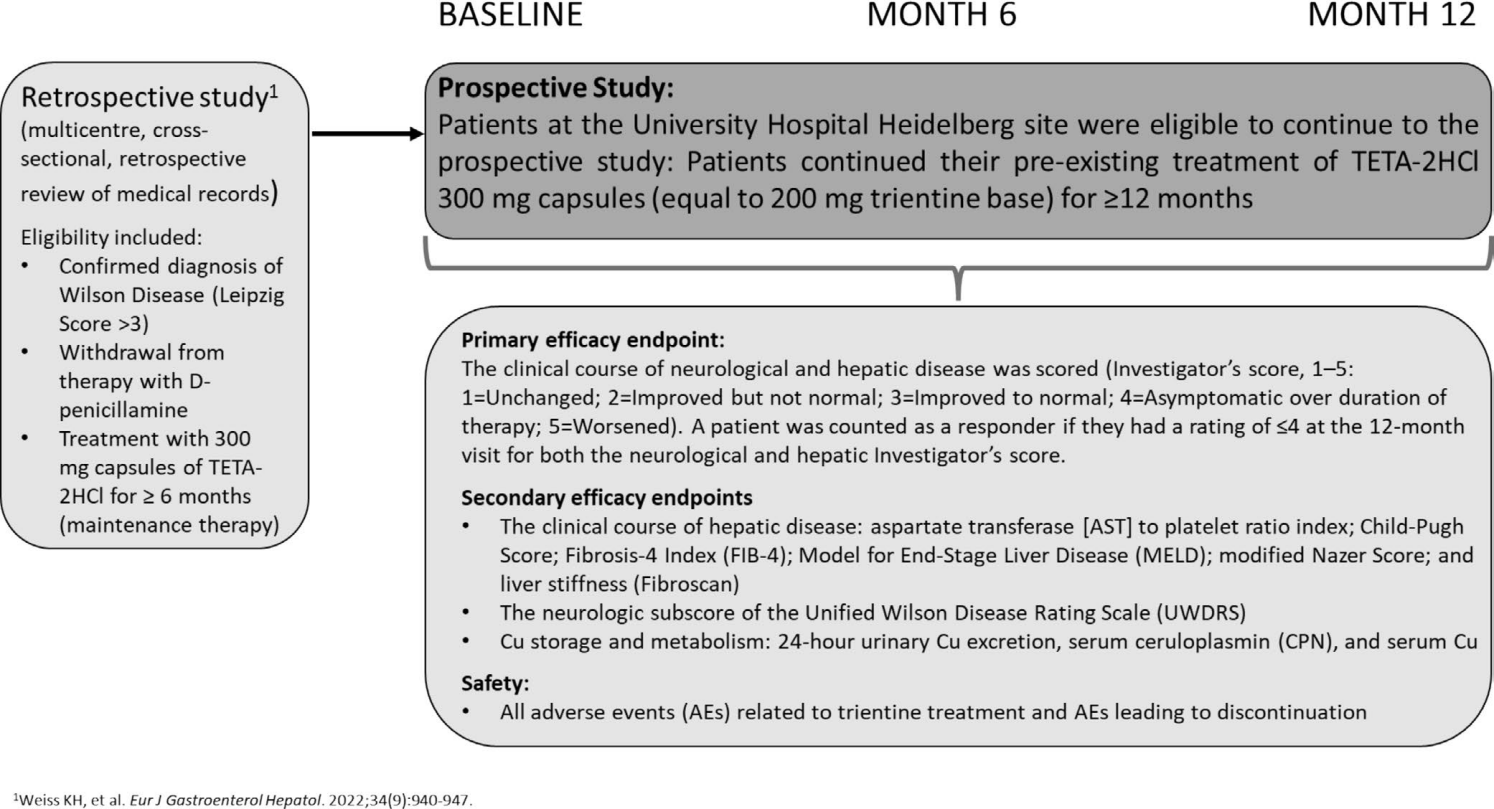
Study design.

This study was a single‐centre, prospective study, of which a retrospective arm has previously been reported [14]. Patients who participated in the retrospective study were eligible to participate in this extension only at the German site (University Hospital Heidelberg) (Figure 1). Prospective assessments were performed at the German site (University Hospital Heidelberg) at Baseline (enrolment) and at 6 and 12 months following the Baseline visit between July 2015 and April 2017. Of note: eligibility for the retrospective part of the study was a confirmed diagnosis of WD (Leipzig Score > 3) and withdrawal from therapy with D‐penicillamine, with subsequent treatment with 300 mg capsules of TETA‐2HCl (equivalent to 200 mg of trientine base) for a period of at least 6 months (maintenance therapy) prior to study inclusion in the prospective arm. The study aimed to assess the efficacy, safety, and tolerability outcomes of chelator‐based treatment after withdrawal of D‐penicillamine and switch to TETA‐2HCl, with a focus on hepatic and especially neurological outcomes. Exclusion criteria were known hypersensitivity to trientine or history of severe anemia. Only data from the prospective arm are reported here.

The study was conducted in accordance with the Declaration of Helsinki and the International Council for Harmonization of Technical Requirements for Pharmaceuticals for Human Use Good Clinical Practices guideline. The Study protocol was approved by the local ethics committees. Written informed consent was obtained from all participants or their legal guardians before inclusion in the study.

### 2.2 Outcome Measures for Efficacy

The primary efficacy outcome was the clinical course of Cu parameters and symptomatic disease progression, where scoring of hepatic disease was based on the Investigator's score at 6 and 12 months after study start. Moreover, Investigator's scoring of neurological disease and subscores was gathered.

Scoring options for the Investigator's score ranged from 1 to 5: 1 (unchanged), 2 (improved but not normal), 3 (improved to normal), 4 (asymptomatic over duration of therapy), and 5 (worsened). A patient was considered a responder if they had a rating of ≤ 4 at the 12‐month visit for both the neurological and hepatic Investigator's score. Patients were counted as non‐responders if they had a rating of 5 for one or both scores at the 12‐month visit, or if they were discontinued from the study for any safety or clinical reason prior to the 12‐month visit. Patients were considered to have stable disease—and thus respond to therapy—for all categories except “worsened”. A similar scoring method had been used previously [13].

Assessment of hepatic disease course included: aspartate transferase (AST) to platelet ratio index (APRI), which is based on AST, gender, and platelet count; Child‐Pugh score, based on international normalized ratio (INR); total bilirubin, albumin, hepatic encephalopathy, and ascites; FIB4 based on age, alanine transferase (ALT), AST, and platelet count; Model for End‐Stage Liver Disease (MELD), based on INR, total bilirubin, creatinine, and sodium; the modified Nazer Score based on albumin, AST, total bilirubin, INR, and leukocytes; and liver stiffness, measured with Fibroscan. Neurological assessment was performed by means of the Unified Wilson Disease Rating Scale (UWDRS).

Assessment of serum and urinary parameters of Copper (Cu) storage and metabolism were 24‐h urinary Cu excretion (24‐h UCE), serum Ceruloplasmin (Cp), serum Cu, and calculated Non‐Ceruloplasmin Bound Copper (NCC; derived from Cu and Cp measurements), compared to values at Baseline. NCC was calculated as NCC (μmol/L) = Serum Copper (μmol/L)–[47.2 × Serum Ceruloplasmin (g/L)] [6].

### 2.3 Outcome Measures for Safety

All AEs related to TETA‐2HCl treatment and AEs leading to study discontinuation were assessed at each available study time point. An AE was defined as being a treatment‐emergent AE (TEAE) if the onset date was on or after the date of the first dosing with TETA‐2HCl.

### 2.4 Statistical Methods

Once enrolled, patients continued in the study for 12 months with data collected at Baseline (enrolment to prospective arm), 6 months, and 12 months. The sample size was a convenience sample based on the number of eligible patients at the participating study centre (all patients from the German centre enrolled in the retrospective part of the study). No formal statistical comparisons were performed, and only descriptive methods were applied. Continuous variables were summarized using the number of patients in the specified category with non‐missing values (n), mean, median, standard deviation, minimum, and maximum values. Categorical variables were summarized using counts and percentages.

## 3 Results

### 3.1 Baseline Characteristics

Overall, 52 patients were enrolled in the prospective part of the study, with data from 51 patients included in the full analysis set (one patient withdrew consent before the first timepoint [month 6]).

Patient demographics are provided in Table 1. The mean (SD) age of patients was 42.3 (14.52) years, and patients were mostly female (67.3%). At Baseline, most patients presented with hepatic (11 patients, 21.6%) or neurological symptoms (12 patients, 23.5%). Patients also presented with mixed disease manifestation (8 patients, 15.7%) and 20 patients were classified as asymptomatic (39.2%) by Investigator opinion. Concomitant medications included zinc as a mineral supplement (4 patients, 7.7%) and zinc acetate (6 patients, 11.5%).

**TABLE 1:** Baseline characteristics.

Parameter (*n* = 52)	Numbers (Mean (SD) and percentages (in %))
Age at Baseline (years)	
Mean (SD)	42.3 (14.52)
Age at initial diagnosis (years)	
Mean (SD)	15.8 (10.53)
Gender, *n* (%)	
Male	17 (32.7)
Female	35 (67.3)
Ethnic origin, *n* (%)	
White Caucasian	51 (98.1)
Black	1 (1.9)
BMI (kg/m^2^)	
Mean (SD)	23.75 (4.516)
Wilson disease presentation, *n* (%)	
Hepatic symptoms	11 (21.6)
Neurological symptoms	12 (23.5)
Mixed disease manifestation	8 (15.7)
Asymptomatic	20 (39.2)
Liver cirrhosis at baseline, *n* (%), all CHILD A	10 (19.6)
Kayser‐Fleischer Ring, *n* (%)	26 (51.0)
Previous treatment with D‐Penicillamin	
Duration of treatment (years), mean (SD; min‐max)	7.25 (4.88; 0.17–13.42)
Previous treatment with TETA‐2HCl at Baseline	
Duration of treatment (years), mean (SD; min‐max)	10.3 (6.5; 0.75–25.60)
Total dose per day during treatment (mg), mean (SD; min‐max)	1377.6 (368.78; 600–2100)

Abbreviations: BMI, body mass index; SD, standard deviation.

### 3.2 Efficacy and Copper Parameters

The mean duration of treatment with TETA‐2HCl in this prospective part of the study was 13.1 months. The mean total dose of TETA‐2HCl salt during treatment was 1377.6 mg per day with a range of 600–2100 mg per day. Dose was noted as mg trientine dihydrochloride salt—not trientine base. Table 2 shows an overview of the copper parameters within this prospective part of the study.

**TABLE 2:** Efficacy and copper parameters.

Parameter	Baseline (mean [SD])	6 months (mean [SD])	12 months (mean [SD])	Change from Baseline to Month 12 (mean [SD])
24‐h UCE [μmol/24 h]	4.24 [2.90]	4.01 [2.09]	4.45 [3.47]	0.21 [1.23]
Serum Copper [μmol/l]	4.51 [3.40]	4.65 [3.00]	4,75 [3.61]	0.24 [1.43]
NCC [μmol/l]	1.04 [0.91]	1.15 [0.93]	0.91 [0.88]	‐ 0.13 [0.31]
Coeruloplasmin [g/l]	0.05 [0.05]	0.07 [0.08]	0.07 [0.06]	0.02 [0.03]

Abbreviations: 24‐h UCE, 24‐h urinary copper excretion; NCC, non‐coeruloplasmin‐bound copper; SD, standard deviation.

Parameters for 24‐h UCE (mean [SD]) were assessed at Baseline (4.24 μmol/24 h [2.90]), Month 6 (4.01 μmol/24 h [2.09]), and Month 12 (4.45 μmol/24 h [3.47]). At the same timepoints, mean (SD) values for serum Cu were 4.51 μmol/L (3.40), 4.65 μmol/L (3.00), and 4.75 μmol/L (3.61), respectively. The NCC levels were at Baseline 1.04 μmol/L (0.91), at Month 6 1.15 μmol/L (0.93), and at Month 12 0.91 μmol/L (0.88). 24‐h UCE increased slightly, whereas NCC decreased, reflecting the inverse correlation of both parameters.

### 3.3 Hepatic Disease Course

The clinical course of hepatic disease is presented in Table 3. Stable hepatic disease was noted for almost all patients at Month 6 (96.1% [95% CI: 86.8, 98.9]) and Month 12 (98% [89.7, 99.7]). No patients required a liver transplant during the course of the study.

**TABLE 3:** Clinical Course of hepatic disease.

	Baseline	6 months	12 months
Hepatic outcome, *n* (%)^a^			
Unchanged	6 (11.8)	18 (35.3)	17 (33.3)
Improved but not normal	10 (19.6)	5 (9.8)	6 (11.8)
Improved to normal	6 (11.8)	1 (2.0)	2 (3.9)
Asymptomatic over duration of therapy	26 (51.0)	25 (49.0)	25 (49.0)
Worsened	3 (5.9)	2 (3.9)	1 (2.0)
Stable hepatic disease, *n* (%) 95% CI^b^		49 (96.1) [86.8, 98.9]	50 (98.0) [89.7, 99.7]
Inadequate hepatic response since last available time point, *n* (%)^a^			
Yes	0	0	1 (2.0)
No	51 (100)	51 (100)	50 (98.0)

*Note:* Patient's stable disease = all categories except “worsened”. Assessments at Baseline were with respect to initiation of treatment with trientine from the retrospective part of the study, the assessments at 6 months and 12 months were with respect to the Baseline of the prospective part.

Abbreviation: CI, confidence interval.

^a^ Percentages were based on the number n of patients with assessment of hepatic outcome/hepatic response.

^b^ Wilson score confidence interval.

At Month 12, almost all patients (98.0%, 95% confidence interval [CI] 89.7, 99.7) were considered responders (rating of ≤ 4 at the 12‐month visit for both the neurological and hepatic Investigator's score). No patients had worsening of hepatic disease at Baseline or Month 6, while 1 (2.0%) patient experienced liver enzyme abnormalities and increasing 24‐h UCE at Month 12. This patient (male, 19 years, primary hepatic manifestation) with worsening of liver disease experienced increasing transaminases (AST/ALT U/L) from Baseline (25/53 U/L) to 6 months (54/181 U/L) and 12 months (63/217 U/L) follow‐up without impairment of liver synthesis or neurological worsening. 24‐h UCE was 6.59 μmol/24 h at Baseline, 8.13 μmol/24 h at 6 months and 6.44 μmol/24 h at 12 months. Compliance evaluation revealed non‐compliance and additional frequent consumption of alcohol, both of which might have contributed to this development. Six months after the end of study compliance was improved and transaminases (40/115 U/L) were again decreasing. Additionally, the total UWDRS Score worsened slightly from Baseline (3 points) to 12 months (4 points) suitable to non‐compliance.

Transaminases and parameters of liver synthesis are presented in Table 4. Overall, biochemical parameters were very similar at all timepoints and revealed a stable disease condition within the 12 months of follow‐up.

**TABLE 4:** Biochemical course of hepatic disease.

	Baseline (mean [SD])	6 months (mean [SD])	12 months (mean [SD])	Change from Baseline to Month 12 (mean [SD])
AST [U/l]	29.50 [15.61]	28.5 [11.51]	30.00 [8.98]	0.5 [10.56]
ALT [U/l]	32.50 [40.96]	32.00 [23.85]	37.00 [29.04]	4.5 [22.40]
GGT [U/l]	38.60 [38.75]	30.00 [6.02]	27.00 [41.55]	−11.6 [13.46]
Bilirubin [U/l]	0.70 [0.50]	0.70 [0.91]	0.70 [1.52]	+ − 0 [0.25]
INR	1.08 [0.08]	1.08 [0.09]	1.06 [0.08]	−0.02 [0.04]
Cholinesterase [kU/l]	6.03 [1.98]	6.02 [1.79]	6.29 [1.73]	0.26 [0.87]
WBC [/nl]	5.60 [2.06]	4.9 [1.90]	5.18 [2.08]	−0.52 [1.52]
Thrombocytes [/nl]	195.00 [86.18]	190.50 [80.68]	196.50 [84.14]	1.5 [55.40]

Abbreviations: ALT, alanine aminotransferase; AST, aspartate aminotransferase; GGT, gamma‐glutamyltransferase; INR, international normalized ratio; SD, standard deviation; WBC, white blood cell count.

Biochemical scores of hepatic disease (Table 5) were similar at all timepoints, with a mean (SD) change from Baseline to Month 12 in AST to platelet ratio index of −0.03 (0.16), FIB‐4 index of −0.01 (0.42), MELD of 0.0 (0.75), modified Nazer score of −0.2 (0.76), and fibroscan stiffness of −0.09 kPa (3.076).

**TABLE 5:** Assessments of clinical course of hepatic disease.

Visit	AST to platelet ratio index (APRI) (mean [SD])	FIB‐4 index (mean [SD])	MELD score (mean [SD])	Sodium‐corrected MELD score (mean [SD])	Modified Nazer score (mean [SD])	Fibroscan: stiffness (kPa) (mean [SD])	Child‐Pugh score (mean [SD])
Baseline	0.52 [0.31]	1.60 [1.58]	7.7 [1.45]	7.7 [1.95]	1.1 [0.83]	7.67 [4.19]	5.1 [0.30]
Month 6	0.49 [0.35]	1.55 [1.58]	7.8 [1.46]	7.9 [1.92]	0.8 [0.71]	8.48 [3.87]	5.1 [0.24]
Change from Baseline to Month 6	−0.03 [0.15]	−0.06 [0.42]	0.1 [0.68]	0.2 [1.98]	−0.3 [0.79]	0.75 [2.52]	0.0 [0.20]
Month 12	0.49 [0.37]	1.59 [1.71]	7.7 [1.66]	8.4 [2.08]	0.9 [0.84]	7.73 [2.81]	5.1 [0.36]
Change from Baseline to Month 12	−0.03 [0.16]	−0.01 [0.42]	0.0 [0.75]	0.7 [2.21]	−0.2 [0.76]	−0.09 [3.08]	0.0 [0.20]

Abbreviations: AST, aspartate transferase; FIB, fibrosis; MELD, model for end‐stage liver disease.

The mean (SD) values for Child‐Pugh score at Baseline were 5.1 at all timepoints (SD 0.24–0.36). At Baseline and at Month 6, all patients received a Child‐Pugh classification of Class A (5–6 points), whereas one (2.0%) patient was classified as Class B (7–9 points) at Month 12.

Laboratory efficacy assessments included the levels of transaminases (ALT, AST, and Gamma‐glutamyl transferase [GGT]), total bilirubin, albumin, INR, and hemoglobin at Month 6 and Month 12. In total, 22 (43.1%) patients at Month 6 and 31 (60.8%) patients at Month 12 had one or more of the liver parameters (ALT, AST, and GGT) outside the normal range. Of these, only one patient had one or more abnormal values considered to be clinically significant (by Investigator opinion).

One or more of the transaminases, total bilirubin, albumin, or INR values were outside the normal range for 29 (56.9%) patients at Month 6 and for 40 (78.4%) patients at Month 12. No other laboratory values were considered clinically significant.

Overall, 9 (17.6%) patients were found to be anemic (hemoglobin below the lower limit of normal) at Month 6 and Month 12.

### 3.4 Neurological Disease Course

The clinical course of neurological disease is presented in Table 6. Stable neurological disease (by Investigator's score) was noted for all patients (95% CI: 93.0%, 100%) at Month 6 and for 98% (95% CI: 89.7%, 99.7%) at Month 12.

**TABLE 6:** Clinical Course of Neurological Disease.

	Baseline	6 months	12 months
Neurological outcome, *n* (%)^a^			
Unchanged	12 (23.5)	31 (60.8)	24 (47.1)
Improved but not normal	8 (15.7)	1 (2.0)	2 (3.9)
Improved to normal	1 (2.0)	0	0
Asymptomatic over duration of therapy	29 (56.9)	19 (37.3)	24 (47.1)
Worsened	1 (2.0)	0	1 (2.0)
Stable neurological disease, *n* (%) 95% CI^b^		51 (100)	50 (98.0)
		[93.0, 100]	[89.7, 99.7]
Inadequate hepatic response since last available time point, *n* (%)^a^			
Yes	1 (2.0)	0	0
No	50 (98.0)	51 (100)	51 (100)

*Note:* Patient's stable disease = all categories except “worsened”. Assessments at Baseline were with respect to initiation of treatment with trientine from the retrospective part of the study, the assessments at 6 months and 12 months were with respect to the Baseline of the prospective part.

Abbreviation: CI, confidence interval.

^a^ Percentages were based on the number n of patients with assessment of hepatic outcome/hepatic response.

^b^ Wilson score confidence interval.

Overall, 19 (37.3%) patients and 24 (47.1%) patients were asymptomatic over the duration of therapy at Month 6 and Month 12, respectively. One (2.0%) patient had a worsening neurological response at Baseline compared to Baseline in the retrospective arm. No patients had an inadequate neurological response at Month 6 or Month 12.

Values for the UWDRS—neurologic subscale assessment—are presented in Table 7. The overall mean (SD) was 11.3 (24.31) at Baseline, 9.7 (23.85) at Month 6, and 8.8 (22.86) at Month 12. The mean (SD) change from Baseline to Month 6 for sub Score I was −0.1 (0.73) and from Baseline to Month 12 was −0.1 (0.81). The mean (SD) change from Baseline to Month 6 for sub Score II was −1.7 (2.67) and from Baseline to Month 12 was −2.5 (4.52).

**TABLE 7:** Unified Wilson Disease Rating Scale: neurologic Subscale.

Visit	Subscore I (mean [SD])	Subscore II (mean [SD])	Total score (mean [SD])
Baseline	1.7 [6.20]	9.6 [18.53]	11.3 [24.31]
Month 6	1.6 [6.13]	8.0 [18.04]	9.7 [23.85]
Change from baseline to Month 6	−0.1 [0.73]	−1.7 [2.67]	−1.8 [3.00]
Month 12	1.7 [6.13]	7.1 [17.04]	8.8 [22.86]
Change from Baseline to Month 12	−0.1 [0.81]	−2.5 [4.52]	−2.5 [4.73]

*Note:* Subscore I—Sum of all items of parts I and II of the UWDRS. Subscore II—Sum of all items of part III of the UWDRS. Total score—Sum of all items of the neurologic subscale of UWDRS.

Abbreviation: SD, standard deviation.

### 3.5 Safety

An overview of TEAEs is provided in Tables S1 and S2. During the study, 42 (80.8%) patients reported TEAEs and 8 (15.4%) patients reported serious TEAEs. Five (9.6%) patients had TEAEs that were considered treatment‐related. The majority of TEAEs were mild or moderate: TEAEs of mild severity were reported by 32 (61.5%) patients and 9 (17.2%) patients reported TEAEs of moderate severity. Only 1 (1.9%) patient reported a severe TEAE (radius fracture) during the prospective part of the study.

The most frequently reported TEAEs were nasopharyngitis (5 [9.6%] patients), cough (4 [7.7%] patients), sinusitis, urinary tract infection, arthralgia, pregnancy, and pruritus (3 [5.8%] patients each). None of the patients withdrew from treatment with TETA‐2HCl due to an AE (one patient withdrew due to personal circumstances), and no serious TEAEs were considered to be treatment‐related.

## 4 Discussion

This prospective study showed that maintenance treatment for WD with TETA‐2HCl was effective, including maintaining stable hepatic and neurologic disease for over a period of 12 months. Additionally, improvement in neurological symptoms continued throughout the trial, suggesting symptomatic improvements may still be reported for an extended time period after the initiation of therapy.

The efficacy and safety profile of TETA‐2HCl has already been demonstrated in several short‐term, mostly retrospective, small‐scale studies in both healthy volunteers and patients with WD [4, 6].

The response observed in this study supports and adds to previous studies, including a recently published, large retrospective study [14], of which this study was the prospective part. In these previous studies, most patients reported stable disease, with several patients reporting continued improvements, and very few patients reporting worsening of symptoms [13, 14].

Among the 51 patients who were treated with TETA‐2HCl in this prospective study, almost all were considered responders. One patient was regarded as a non‐responder due to the worsening of hepatic disease status at Month 12.

Stable hepatic disease was noted for almost all patients throughout the study, reflected in stable AST/ALT levels and stable biochemical hepatic scores (APRIm FIB.4, modified Nazer, Child Pugh score). Surprisingly, UWDRS scores improved throughout the duration of the study. This indicates a continuing improvement in neurological symptoms, even during maintenance therapy in WD patients. This supports previous reports that neurological impairment—albeit slowly—can still improve in patients following maintenance chelator‐based treatment [18].

Biochemical and clinical assessments also showed improvement or stability for the duration of the study; serum and urinary parameters of Cu storage and metabolism were similar at Baseline, Month 6, and Month 12. 24‐h UCE was > 4.0 μmol/24 h, which is within expected values for WD patients (> 1.6 μmol/24 h) [2] and within guideline recommended range for patients receiving chelation therapy (3–8 μmol/24 h) [5]. Similarly, in a randomized, open‐label, non‐inferiority, Phase 3 trial of trientine in comparison to D‐penicillamine, 24‐h UCE was also within guideline ranges for patients treated with either D‐penicillamine or TETA‐4HCl [17]. Additionally, NCC was within normal levels (0.7–2.4 μmol/L), as expected with effective maintenance treatment [5] and declined over 12 months from 1.04 μmol/L at Baseline to 0.91 μmol/L at Month 12  while the 24‐h UCE increased slightly within the 12 months.

In the current study, patients had been previously treated with D‐penicillamine for 7.25 years (SD 4.88; min 0.17–max 13.42), followed by treatment with TETA‐2HCl for at least a minimum of 6 months prior to study inclusion; as evidenced by serum and urinary parameters of Cu storage and metabolism within the normal range at Baseline, all patients should therefore have completed the “decoppering” phase prior to study entry [8]. It is therefore not unexpected for many patients to report stable hepatic and neurologic disease throughout the study. However, the continued improvement in neurological symptoms reported by several patients suggests that the full benefits of removal of excess Cu may be ongoing for an extended time period. Continued adherence to treatment regimens and adequate dosing of Cu chelators is therefore vital for patients to gain the greatest benefits from their treatment.

Limitations of the trial include the open‐label study design and absence of a comparator arm or the enrolment of treatment‐naive patients. However, proportions of patients improving under maintenance therapy with trientine were consistent with previous trials [13, 14]. The safety profile and number of AEs observed during this trial were consistent across previous studies with TETA‐2HCl [4, 6, 13, 14, 15, 16].

In conclusion, this prospective study assessing long‐term outcomes of chelator‐based treatment with TETA‐2HCl in WD demonstrated that maintenance treatment with TETA‐2HCl was effective in maintaining stable hepatic and neurologic disease. Additionally, improvement in neurological symptoms was reported throughout the trial, suggesting continued improvements may still be reported for an extended time period. Overall, TETA‐2HCl was generally effective, safe, and well tolerated.

## Ethics Statement

All procedures performed in studies involving human participants were in accordance with the ethical standards of the institutional and/or national research committee and with the 1964 Helsinki declaration and its later amendments or comparable ethical standards.

## Consent

Informed consent was obtained from all individual participants included in the study.

## Conflicts of Interest

I.M. advises for Univar Solutions and Orphalan, I.M. received a travel grant from Univar Solutions and Alexion. I.M. is the principal investigator in clinical trials from Univar solutions and Alexion. C.K. and V.A. are employees of Univar Solutions. K.H.W. consults for Orphalan, Univar, Alexion, Desitin, Keyproteo, Regeneron, Ultragenyx, Pfizer, and Vivet therapeutics. K.H.W. is a speaker for Abbvie, Alexion, and Falk.

## Supporting information


Data S1.

## Data Availability

The data that support the findings of this study are available from the corresponding author upon reasonable request.
