# Supplementary material for: Prospective Study to Assess Long‐Term Outcomes of Chelator‐Based Treatment With Trientine Dihydrochloride in Patients With Wilson Disease
Source: JGH Open. 2025 Mar 17;9(3):e70114. doi: 10.1002/jgh3.70114 (PMC11914392; doi:10.1002/jgh3.70114)
Supplement: Supplementary file 1 — Data S1. [file JGH3-9-e70114-s001.doc]

Title: Prospective Study to Assess Long-term Outcomes of Chelator-based Treatment with Trientine Dihydrochloride in Patients with Wilson Disease

**Authors: Isabelle Mohr^1^, Carlot Kruse^2^, Verena Aliane^2^, Karl Heinz Weiss^3^**

Supplementary Table 1. Summary of adverse events

|  | **Trientine (N=52)**  **n (%)** |
| --- | --- |
| Patients with any SAE | 8 (15.4) |
| Patients with any TEAE | 42 (80.8) |
| Patients with any serious TEAE | 8 (15.4) |
| Patients with any trientine-related TEAEs | 5 (9.6) |

AE, adverse event, SAE, serious adverse event; TEAE, treatment emergent adverse event

Supplementary Table 2. Summary of treatment-emergent adverse events in ≥3% of patients by system organ class and preferred term

| System Organ Class  Preferred Term | Trientine  (N=52) n (%) |
| --- | --- |
| Patients with any TEAE | 42 (80.8) |
| Blood and lymphatic system disorders | 5 (9.6) |
| Anaemia | 2 (3.8) |
| Pancytopenia | 2 (3.8) |
| Gastrointestinal disorders | 8 (15.4) |
| Abdominal discomfort | 2 (3.8) |
| Hepatobiliary disorders | 2 (3.8) |
| Gallbladder polyp | 2 (3.8) |
| Infections and infestations | 17 (32.7) |
| Nasopharyngitis | 5 (9.6) |
| Sinusitis | 3 (5.8) |
| Urinary tract infection | 3 (5.8) |
| Tonsillitis | 2 (3.8) |
| Viral infection | 2 (3.8) |
| Investigations | 3 (5.8) |
| Hepatic enzyme increased | 2 (3.8) |
| Musculoskeletal and connective tissue disorders | 9 (17.3) |
| Arthralgia | 3 (5.8) |
| Back pain | 2 (3.8) |
| Nervous system disorders | 5 (9.6) |
| Headache | 2 (3.8) |
| Pregnancy, puerperium and perinatal conditions | 3 (5.8) |
| Pregnancy | 3 (5.8) |
| Renal and urinary disorders | 2 (3.8) |
| Renal cyst | 2 (3.8) |
| Respiratory, thoracic and mediastinal disorders | 9 (17.3) |
| Cough | 4 (7.7) |
| Skin and subcutaneous tissue disorders | 4 (7.7) |
| Pruritus | 3 (5.8) |

TEAE, treatment emergent adverse event
